# *Pteroceraphron* Dessart new to the USA (Hymenoptera: Ceraphronoidea)

**DOI:** 10.3897/BDJ.4.e9261

**Published:** 2016-12-09

**Authors:** István Mikó, Lubomír Masner, Andrew Robert Deans

**Affiliations:** ‡ Pennsylvania State University, University Park, United States of America; § Canadian National Collection of Insects, Arachnids and Nematodes, Ottawa, Canada

**Keywords:** Ceraphronidae, marginal cilia, club, USA

## Abstract

**Background:**

*Pteroceraphron* is a monotypic genus that can be recognized by its unique, lanceolate wing shape. Until now the only described species, *Pteroceraphron mirabilipennis* Dessart 1981, was known only from specimens collected in Canada.

**New information:**

Here, for the first time, we report *Pteroceraphron mirabilipennis* Dessart 1981 specimens collected in the USA. We also provide an extended diagnosis.

## Introduction

**Figure 1.**
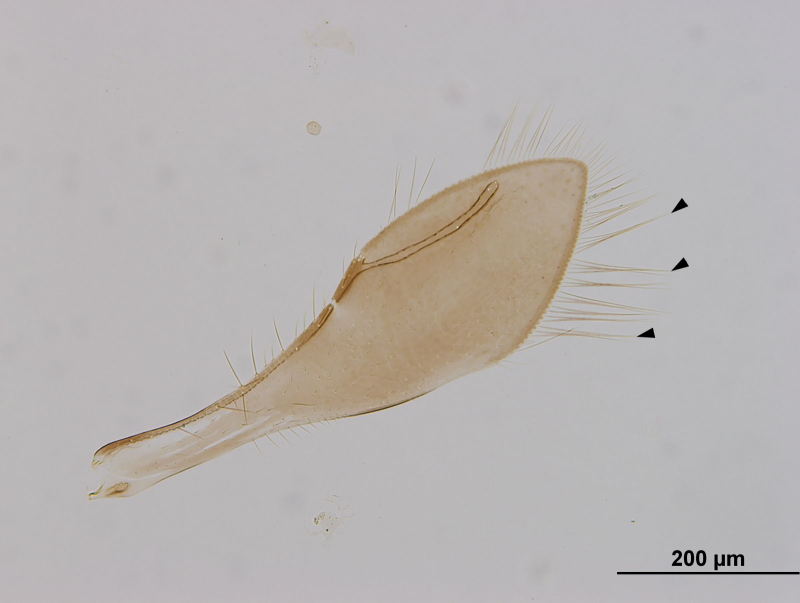
Brightfield image showing the fore wing of *Pteroceraphron mirabilipennis* Dessart 1981. Arrows point to elongate marginal cilia.

The superfamily Ceraphronoidea represents more than 600 described species in 32 genera worldwide (Johnson and Musetti 2004). These wasps are minute, measuring 0.5–4.3 mm in body length, and can be easily diagnosed by their reduced wing venation. The fore wing has only one relatively thick and distally interrupted marginal vein and a curved stigmal vein (Fig. 1). Ceraphronoids are mostly parasitoids of entomophagous insects with an exceptionally large host range that encompasses at least eight insect orders: Coleoptera, Diptera, Hemiptera, Hymenoptera, Mecoptera, Neuroptera, Thysanoptera, and Trichoptera (Mikó et al. 2013). The family Ceraphronidae is one of the most commonly collected microhymenopterans (Martínez de Murgía et al. 2001, Schmitt 2004). External somatic morphology is relatively monotonous and the morphological diversity of the male genitalia is extraordinary (Mikó et al. 2013, Ernst et al. 2013). *Pteroceraphron* stands apart from other ceraphronid genera by having lanceolate fore wings ornamented with elongate marginal setae (Fig. 1). The genus is represented by only one Nearctic species, *Pteroceraphron mirabilipennis*, described by Paul Dessart (Dessart 1981) based on two female specimens collected in Ontario, Canada. Here we report on specimens collected in the United States that extend the range of *Pteroceraphron mirabilipennis* approximately 1,500 km southwards. 

## Materials and methods

Specimens were borrowed from the Canadian National Collection of Insects and Arachnids (CNC). Images were taken with an Olympus CX41 compound microscope and DP71 digital camera. Images were stacked with a Combine Z4 software Hadley 2016) and modified with Adobe Photoshop CS4® using the "auto level" and "unsharp mask" tools. Taxonomic nomenclature, specimen information, OTU concepts were compiled in mx (http://purl.org/NET/mx-database). Figures were deposited in Figshare (doi:10.6084/m9.figshare.3846591).

## Taxon treatments

### Pteroceraphron mirabilipennis

Dessart 1981

Pteroceraphron mirabilipennis Dessart 1981

#### Materials

- **Type status:** Other material. **Occurrence:** recordedBy: BRC HYM. TEAM; individualID: NCSU 0028995; sex: female; **Taxon:** family: Ceraphronidae; genus: Pteroceraphron; specificEpithet: mirabilipennis; **Location:** country: USA; stateProvince: Virginia; county: Northampton; verbatimLocality: Northampton Co. 7km S Jackson; **Identification:** identifiedBy: István Mikó; dateIdentified: 2010; **Event:** eventDate: VIII-IX, 1987; **Record Level:** institutionCode: CNC
- **Type status:** Other material. **Occurrence:** recordedBy: BRC HYM. TEAM; individualID: NCSU 228994; sex: female; **Taxon:** family: Ceraphronidae; genus: Pteroceraphron; **Location:** country: USA; locality: Georga, McINtosh Co. Sapelo Island; verbatimLatitude: 31.38N; verbatimLongitude: 81.28W; **Identification:** identifiedBy: István Mikó; dateIdentified: 2015; **Event:** verbatimEventDate: VI-VII. 1987; **Record Level:** institutionID: CNC
- **Type status:** Other material. **Occurrence:** recordedBy: D.C. Darling; individualID: NCSU 28992; sex: female; **Taxon:** family: Ceraphronidae; genus: Pteroceraphron; **Location:** country: USA; locality: IN, Porter, Cowles Bog; verbatimLatitude: 41.65N; verbatimLongitude: 87.1W; **Identification:** identifiedBy: Dessart Paul; **Event:** verbatimEventDate: 04.VIII.1981; **Record Level:** institutionCode: CNC
- **Type status:** Paratype. **Taxon:** family: Ceraphronidae; genus: Pteroceraphron; **Location:** country: Canada; locality: Ontario, St. Lawrence national Park; **Identification:** identifiedBy: P. Dessart; **Record Level:** modified: 2010-09-24 10:27:52
- **Type status:** Other material. **Occurrence:** recordedBy: M. Sharkey; individualID: PSUC_FEM 000096175; sex: female; **Taxon:** family: Ceraphronidae; genus: Pteroceraphron; **Location:** country: USA; stateProvince: KY; verbatimLocality: KY: Lexington Co.; verbatimLatitude: 37°58'39''N; verbatimLongitude: 84°24'59"W; **Identification:** identifiedBy: István Mikó; dateIdentified: 2015; **Event:** verbatimEventDate: 04-11.vii.2004; **Record Level:** institutionID: CNC
- **Type status:** Holotype. **Taxon:** scientificName: *Pteroceraphron mirabilipennis* Dessart 1981; **Location:** country: Canada; locality: Ontario, St. Lawrence national Park
- **Type status:** Other material. **Occurrence:** recordedBy: BRC HYM. TEAM; individualID: NCSU 28901; sex: female; **Taxon:** family: Ceraphronidae; genus: Pteroceraphron; **Location:** country: USA; locality: Georga, McINtosh Co. Sapelo Island; verbatimLatitude: 31.38N; verbatimLongitude: 81.28W; **Identification:** identifiedBy: István Mikó; **Event:** verbatimEventDate: VI-VII. 1987; **Record Level:** institutionID: CNC
- **Type status:** Other material. **Occurrence:** recordedBy: BRC HYM. TEAM; individualID: NCSU 28900; sex: female; **Taxon:** family: Ceraphronidae; genus: Pteroceraphron; **Location:** country: USA; locality: Georga, McINtosh Co. Sapelo Island; verbatimLatitude: 31.38N; verbatimLongitude: 81.28W; **Identification:** identifiedBy: István Mikó; dateIdentified: 2015; **Event:** verbatimEventDate: VI-VII. 1987; **Record Level:** institutionID: CNC
- **Type status:** Other material. **Occurrence:** recordedBy: N.F. Johnson; individualID: PSUFEM_13529; **Location:** country: USA; county: Franklin; verbatimLocality: USA: Ohio, Franklin Co. patch of praire on Kinnear Rd. across the street from the OSU Museum of Biol. Div.; **Event:** samplingProtocol: YPT; eventDate: 02.vii.1999; **Record Level:** institutionID: Ohio State University Insect Collection; collectionID: OSU
- **Type status:** Other material. **Occurrence:** recordedBy: N.F. Johnson; individualID: PSUFEM_13459; **Location:** country: USA; county: Franklin; verbatimLocality: USA: Ohio, Franklin Co. patch of praire on Kinnear Rd. across the street from the OSU Museum of Biol. Div.; **Event:** samplingProtocol: YPT; eventDate: 02.vii.1999; **Record Level:** institutionID: Ohio State University Insect Collection; collectionID: OSU
- **Type status:** Other material. **Occurrence:** recordedBy: J. T. Becker; **Location:** country: USA; stateProvince: Missouri; locality: Williamsville; **Event:** samplingProtocol: Malaise Trap; eventTime: vi.1987; **Record Level:** institutionID: CAS (California Academy of Sciences)

#### Diagnosis

**Figure 2.**
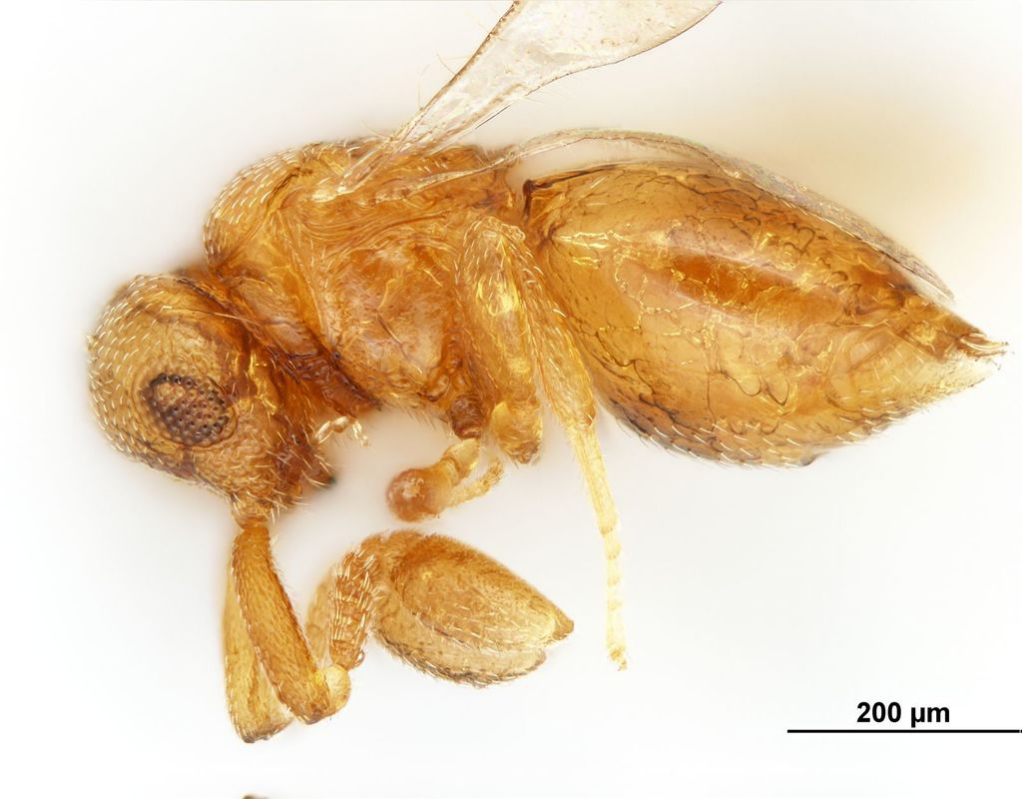
Brightfield image showing the lateral habitus of *Pteroceraphron mirabilipennis* Dessart 1981.

**Figure 3.**
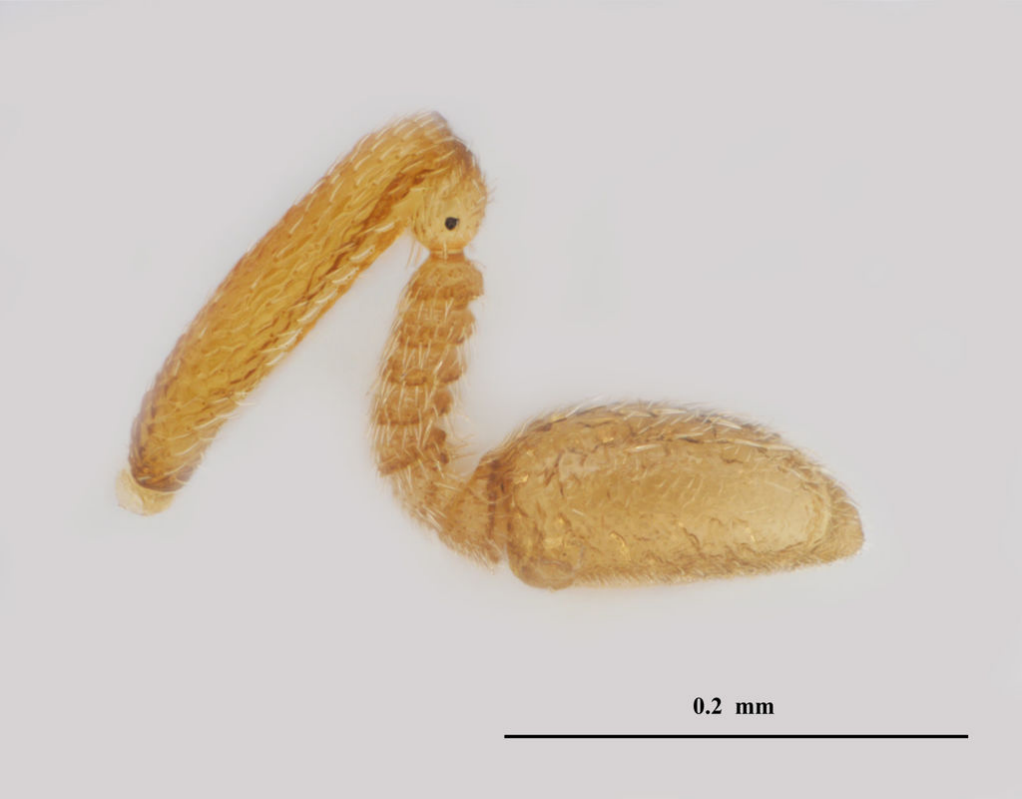
Brightfield image showing the female antenna of *Pteroceraphron mirabilipennis* Dessart 1981.

**Figure 4.**
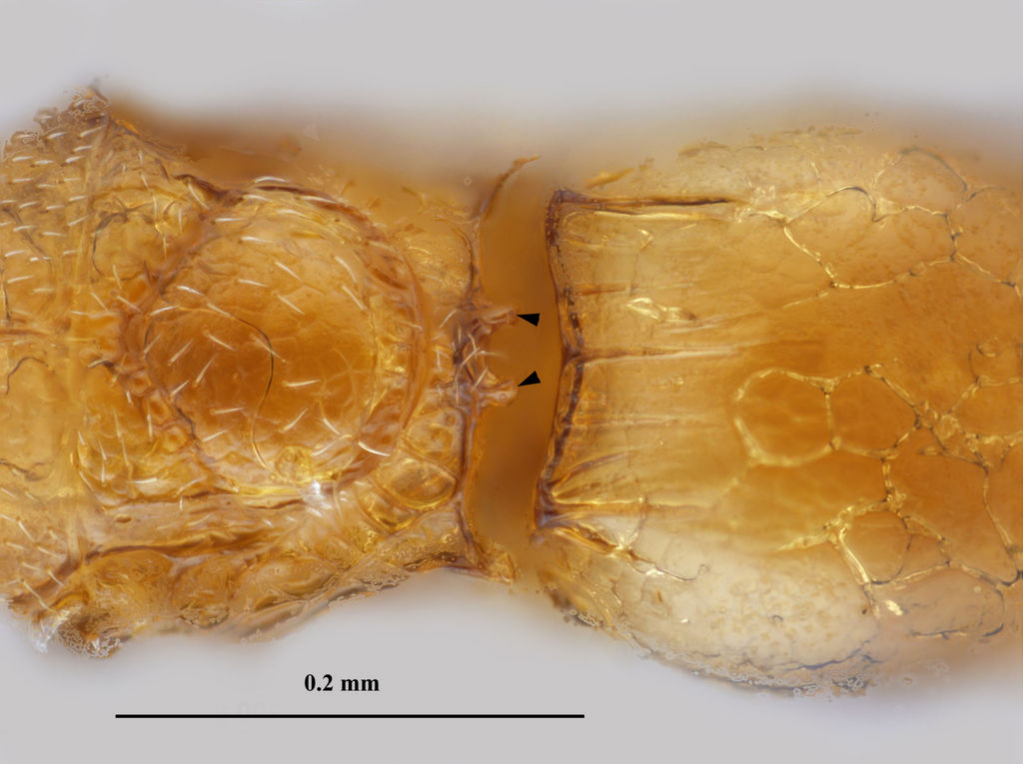
Brightfield image showing the posterior mesosoma and anterior metasoma of *Pteroceraphron mirabilipennis* Dessart 1981. Arrows point to bifurcated anteromedian process of the propodeum-metanotum complex.

*Pteroceraphron mirabilipennis* differs from all other ceraphronoid wasps in the presence of elongate marginal setae (Fig. 1) on the posterior margin of the lanceolate fore wing (Fig. 1). Beside the diagnostic wing characters, the combination of the enlarged last flagellomere (Figs 2, 3), the bifurcated anteromedian process of the propodeum-metanotum complex (Fig. 4), and the presence of three longitudinal carinae on the first metasomal tergum (Fig. 4) make this species easy to separate from other ceraphronids, even if the wings are absent.

## Discussion

These records indicate that *Pteroceraphron*, a distinctive and easily diagnosed taxon, is widespread across eastern North America. Yet virtually nothing about their range, hosts, morphology, or other aspects of their biology has been published. This situation underscores the lack of research on Ceraphronoidea in the Nearctic, a region that was deliberately ignored by past researchers (see Dessart 1997, Dessart 2001).

## Supplementary Material

XML Treatment for Pteroceraphron mirabilipennis
